# Fortified Balanced Energy-Protein Supplementation, Maternal Anemia, and Gestational Weight Gain: A Randomized Controlled Efficacy Trial among Pregnant Women in Rural Burkina Faso

**DOI:** 10.1093/jn/nxac171

**Published:** 2022-07-30

**Authors:** Giles Hanley-Cook, Laeticia C Toe, Kokeb Tesfamariam, Brenda de Kok, Alemayehu Argaw, Anderson Compaoré, Moctar Ouédraogo, Trenton Dailey-Chwalibóg, Patrick Kolsteren, Carl Lachat, Lieven Huybregts

**Affiliations:** Department of Food Technology, Safety and Health, Faculty of Bioscience Engineering, Ghent University, Ghent, Belgium; Department of Food Technology, Safety and Health, Faculty of Bioscience Engineering, Ghent University, Ghent, Belgium; Nutrition and Metabolic Diseases Unit, Health Sciences Research Institute (IRSS), Bobo-Dioulasso, Burkina Faso; Department of Food Technology, Safety and Health, Faculty of Bioscience Engineering, Ghent University, Ghent, Belgium; Department of Food Technology, Safety and Health, Faculty of Bioscience Engineering, Ghent University, Ghent, Belgium; Department of Food Technology, Safety and Health, Faculty of Bioscience Engineering, Ghent University, Ghent, Belgium; AFRICSanté (Health Research and Expertise Training Agency for Africa), Bobo-Dioulasso, Burkina Faso; AFRICSanté (Health Research and Expertise Training Agency for Africa), Bobo-Dioulasso, Burkina Faso; Department of Food Technology, Safety and Health, Faculty of Bioscience Engineering, Ghent University, Ghent, Belgium; Department of Food Technology, Safety and Health, Faculty of Bioscience Engineering, Ghent University, Ghent, Belgium; Department of Food Technology, Safety and Health, Faculty of Bioscience Engineering, Ghent University, Ghent, Belgium; Department of Food Technology, Safety and Health, Faculty of Bioscience Engineering, Ghent University, Ghent, Belgium; Poverty, Health, and Nutrition Division, International Food Policy Research Institute (IFPRI), Washington, DC, USA

**Keywords:** anemia, balanced energy-protein, Burkina Faso, gestational weight gain, iron–folic acid, multiple micronutrients, randomized controlled trial

## Abstract

**Background:**

Anemia and suboptimal gestational weight gain (GWG) are associated with adverse maternal and birth outcomes. Limited research indicates that balanced energy-protein (BEP) supplements reduce the incidence of inadequate GWG.

**Objectives:**

We assessed the efficacy of a micronutrient-fortified BEP supplement on the secondary outcomes of anemia, GWG, GWG rate, and GWG in relation to the Institute of Medicine (IOM)’s recommendations, as compared with an iron–folic acid (IFA) tablet.

**Methods:**

We conducted a randomized controlled trial in Burkina Faso, among pregnant women (15–40 y old) enrolled at <21 weeks of gestation. Women received either BEP and IFA (intervention) or IFA (control). Hemoglobin (g/dL) concentrations were measured at baseline and the third antenatal care visit (ANC), whereas maternal weight was measured at baseline and all subsequent ∼7-weekly ANCs. GWG (kg) was calculated as a woman's last weight measurement (at ∼36 weeks of gestation) minus weight at enrollment, whereas GWG rate (kg/wk) was GWG divided by the time between the first and last weight measurements. GWG adequacy (%) was computed as GWG divided by the IOM's recommendation. Binary outcomes included severely inadequate, inadequate, and excessive GWG. Statistical analyses followed the intention-to-treat principle. Linear regression and probability models were fitted for the continuous and binary outcomes, respectively, adjusting for baseline measurements.

**Results:**

Women in the BEP group tended to have higher, but nonsignificantly different, GWG (0.28 kg; 95% CI: −0.05, 0.58 kg; *P* = 0.099). Furthermore, there were no significant differences in prenatal anemia prevalence, GWG rate, GWG adequacy, or incidence of inadequate or excessive GWG. Findings were robust to model adjustments and complete case and per protocol analyses.

**Conclusions:**

This trial does not provide evidence that fortified BEP supplementation reduces maternal anemia or increases GWG, as compared with IFA. In conjunction with the small, but positive, effects of maternal BEP supplementation on birth outcomes, our findings warrant the investigation of additional biochemical and postnatal outcomes.

This trial was registered at clinicaltrials.gov as NCT03533712.

## Introduction

Maternal gestational weight gain (GWG) is a cumulative measure reflecting the altering physiology of the mother (fat and fat-free mass deposition, as well as breast tissue, blood volume, and extracellular fluid expansion), the gravid uterus, the placental weight, and the developing fetus (fat and fat-free mass, as well as the amniotic fluid accretion) (1). Suboptimal GWG, a modifiable factor by both preconception and antenatal care (2, 3), has been related to adverse maternal and birth outcomes. Low GWG is associated with an increased prevalence of low birth weight and small-for-gestational age (SGA) at birth, and greater GWG is associated with a higher prevalence of large-for-gestational age and macrosomia (4–6). Furthermore, both lower and higher GWG have been associated with an increased risk of preterm birth (4). In addition, women who gain excessive weight during pregnancy may experience various adverse maternal outcomes, including the progression of gestational diabetes, complications during labor, increased prevalence of cesarean delivery, and subsequent maternal postpartum weight retention, obesity, and cardiovascular disorders (7, 8). However, high-quality interventional or epidemiologic GWG data remain scarce for low- and middle-income countries (LMICs) (9).

In 2009, the Institute of Medicine (IOM), now called the National Academy of Medicine, re-examined their GWG recommendations, stratified by prepregnancy maternal BMI (in kg/m^2^) (1). At present, no universally accepted GWG references exist (10); therefore, the IOM's recommendations, which are based entirely on studies conducted in high-income countries, are widely used in LMICs. Two recent studies using data from Demographic and Health Surveys estimated that the mean total GWG in sub-Saharan Africa was 6.5 kg (95% CI: 6.0, 7.0 kg) (11) or 6.6 kg (95% CI: 3.4, 9.9 kg) (12), which is about half the IOM's minimum recommendations of 11.5 kg for normal-weight and 12.5 kg for underweight women. Attaining optimal GWG may not only improve immediate newborn outcomes, but may also confer many potential long-term benefits. Prior evidence has shown that optimal GWG is associated with decreased risks of infant mortality, childhood overweight, as well as adulthood obesity (13, 14).

To date, 2 systematic reviews have assessed the effectiveness of prenatal nutritional supplements on GWG. A 2018 Cochrane review indicated no difference in GWG, when small quantity lipid-based nutrient supplements (SQ-LNSs) were compared against iron–folic acid (IFA) or multiple micronutrient (MMN) supplementation; however, IFA was more effective than SQ-LNSs in terms of reducing maternal anemia (15). Similarly, a 2015 Cochrane review of the limited number of (fortified) balanced energy-protein (BEP) supplementation trials reported a null effect on GWG, but highlighted the very-low-quality evidence (16). Two other systematic reviews have suggested that prenatal anemia is associated with an increased risk of adverse pregnancy outcomes, such as preterm birth and low birth weight (17, 18). To our knowledge, no study has assessed the effect of MMN-fortified BEP supplements on maternal anemia, because most prior BEP trials aimed to cover energy and macronutrient requirements only (19).

Using data from the MIcronutriments pour la SAnté de la Mère et de l'Enfant (MISAME)-III randomized controlled trial (RCT) among pregnant women in rural Burkina Faso (20), we assessed the efficacy of prenatal fortified BEP supplementation, as compared with IFA tablets (i.e., the standard of care), on maternal anemia, absolute GWG (both prespecified secondary study outcomes), GWG rate, and adequacy of GWG based on the IOM's recommendations (1). The results from this analysis will contribute to understanding the previously reported modest effects of fortified BEP supplements on birth outcomes observed in the MISAME-III trial (e.g., 50.1 g increase in birth weight; 95% CI: 8.11, 92.0 g) (21), and inform on effective prenatal nutritional interventions to achieve optimal GWG in LMICs.

## Methods

Our research was reported using the CONSORT 2010 checklist (22).

### Study setting

The prenatal phase of the MISAME-III study (NCT03533712) was conducted between the first enrollment on 30 October, 2019 and the last delivery on 7 August, 2021 in the catchment areas of 6 rural health centers of the health district of Houndé, Tuy Province, in the Hauts-Bassins region of Burkina Faso. In the preceding MISAME-I (23) and MISAME-II (24) RCTs, 48.4% and 45.5% of pregnant women were anemic [hemoglobin (Hb) < 11 g/dL] at baseline, respectively. Malaria transmission is perennial, with seasonal variations. The usual diet during pregnancy is nondiverse (25), predominantly maize-based with a complement of leafy vegetables (26), and consequently dietary micronutrient intakes are inadequate to cover the Estimated Average Requirements (EARs) (27). Moreover, among a subsample of MISAME-III women, the mean daily energy intake of the base diet (i.e., excluding supplements) was estimated to be ∼1940 kcal in both trial arms at the end of the preharvest season (27).

### Study design, participants, and enrollment procedures

The MISAME-III protocol was published previously (20). In brief, the study was a community-based, nonblinded, individually randomized 2 × 2 factorial RCT, with directly observed daily supplement intake.

Women aged between 15 and 40 y and living in the study villages were identified through a census in the study area (*n* = 10,165). A network of 142 locally trained community support staff visited all eligible women at their homes every 5 wk to identify pregnancy early, by screening for self-reported amenorrhea. Women suspected to be pregnant were referred to the health center for a urine pregnancy test. Once gestation was preliminarily confirmed, the MISAME-III study purpose and procedures were explained in the local language: Bwamu, Mooré, or Dioula. Before randomization, we excluded women who intended to leave the study area during their pregnancy, planned to deliver outside the study area, or mothers who had a peanut allergy.

After written informed consent was obtained, women (pregnancy not yet confirmed by an ultrasound) were randomly assigned to receive either a daily fortified BEP supplement and IFA tablet (intervention group) or a daily IFA tablet alone (control group) during pregnancy. The stratified randomization scheme per health center was generated by an external research analyst before the start of the study with Stata version 15.1 (StataCorp), in permuted blocks of 8 (4 control, 4 intervention). The allocation was coded with the letter A for the prenatal control arm and the letter B for the prenatal intervention arm. Randomization codes were concealed in sequentially numbered sealed opaque envelopes by project employees, who were not in direct contact with enrolled women. The project midwives, who enrolled participants, assigned women to a trial arm by drawing a sealed envelope containing the A/B letter code. MISAME-III enrollment ran from 30 October, 2019 to 12 December, 2020. Within 14 d of enrollment, a woman's pregnancy was definitively confirmed by an ultrasound. Gestational age (GA) was estimated by measuring crown–rump length (7–13 weeks of gestation) or by calculating the mean of 3–4 measurements: biparietal diameter, head circumference, abdominal circumference, and femur length (12–26 weeks of gestation) (23). Postrandomization, we excluded nonpregnant women, mothers with a GA ≥ 21 completed weeks, and multifetal pregnancies (i.e., not meeting the a priori–defined study inclusion criteria) (28).

Trained village-based project workers visited 10–25 pregnant women per day to ensure the directly observed intake of BEP supplements and IFA tablets. When women had a short and scheduled absence from home, BEP supplements and IFA tablets were given to the mother in advance (thus, counted as nonobserved intakes for the respective days). The home visitors also encouraged pregnant women to attend ≥4 scheduled ANC visits every ∼7 wk. All serious adverse events (e.g., miscarriage and stillbirth) were recorded on a case-by-case basis, and verbal autopsies were conducted by the MISAME-III physician for maternal or infant deaths that occurred outside a health center.

Because the fortified BEP supplement and IFA tablet were identifiable, it was not possible to blind study mothers or community support staff. However, the RCT's physician and midwives responsible for measuring the prenatal secondary maternal outcomes might be deemed partially blinded (although access was permitted to the allocation code in the enrollment file). Researchers who managed, cleaned, and analyzed MISAME-III data were not blinded.

### Study supplements

In 2016, the Bill & Melinda Gates Foundation convened an expert group to recommend the optimal nutritional composition of the BEP supplement (29). In a formative study, the most preferred and suitable MMN-fortified BEP supplement was selected for administration in the MISAME-III efficacy trial (30, 31). The BEP supplement is an LNS in the form of an energy-dense peanut paste fortified with MMNs. The BEP is made by Nutriset and is ready-to-consume, does not require a cold chain, and has a long shelf life. On average, the 72-g fortified BEP provided 393 kcal and consisted of 36% lipids, 20% protein, and 32% carbohydrates. Furthermore, the MMN content alone covered at least the IOM's daily EARs of micronutrients for pregnant women, except for calcium, phosphorus, and magnesium (32). **Supplemental Table 1** provides the complete nutritional composition of the MMN-fortified BEP.

Women in the intervention group daily received a fortified BEP supplement and an IFA tablet {65 mg Fe (form: FeH_2_O_5_S) and 400 μg folic acid [form: C_19_H_19_N_7_O_6_; Tolerable Upper Intake Level from fortified food or supplements, not including folate from food: 1000 μg/d (33)]}, whereas women in the control group daily received an IFA tablet only (Sidhaant Life Sciences), in accordance with Burkina Faso's national health protocol (i.e., standard of care). Following Burkinabe guidelines, all enrolled women received malaria prophylaxis (3 oral doses of sulfadoxine-pyrimethamine) at the relevant ANC visits.

### Data collection and measures

At enrollment (i.e., first ANC visit), we measured maternal height, weight, midupper arm circumference (MUAC) in duplicate, and Hb concentration. Maternal weight and MUAC were measured again, in duplicate, at each subsequent ANC visit. Hb concentration was assessed again between 19 and 34 weeks of gestation (i.e., third ANC visit). Furthermore, a comprehensive socioeconomic and demographic questionnaire was administered at baseline (20).

Maternal height was measured to the nearest 1 cm using a ShorrBoard® Infant/Child/Adult (Weigh and Measure) and weight to the nearest 100 g with a Seca 876 scale (Seca); and the accuracy of the scales was verified weekly. Maternal MUAC was measured to the nearest 1 mm using a Seca 212 measuring tape (Seca). Pregnant women's Hb concentration was measured by spectrophotometry with a HemoCue® Hb 201+ (HemoCue); and a weekly calibration check was made with the use of a HemoCue Control Cuvette. The study's physician performed transabdominal ultrasound fetal biometry within 2 wk of enrollment. Pregnancy was confirmed and GA was estimated using a portable diagnostic imaging and full-color, flow-mapping SonoSite M-Turbo (Fujifilm SonoSite Inc.). Concurrently, maternal subscapular and tricipital skinfold measurements were taken in triplicate using a Harpenden caliper.

MISAME-III data were collected using SurveySolutions version 21.5 (The World Bank) on tablets by the project physician and midwives; these data were transferred to a central Ghent University server weekly. Questionnaire assignments were sent once a week to the field team including preloaded data collected at a previous ANC visit to lower the amount of incorrect data. Furthermore, we programmed generic validation codes to avoid the entry of implausible values and to improve the quality of data collection in the field. In addition, biweekly data quality checks were conducted and missing or inconsistent data were sent back to the field for revision. The quality of ultrasound images and estimation of GA were checked for >10% of the examinations regularly by an external gynecologist, using a quality checklist and scoring sheet. The trained project workers collected daily data on fortified BEP and IFA compliance in both prenatal study arms via smartphone-assisted personal interviewing programmed in CSPro version 7.3.1 (U.S. Census Bureau, ICF, and Serpro) . Six supervisors performed monthly lot quality assurance sampling schemes of each home visitor's work on a random day (34).

All data collection forms are available on the study's website: https://misame3.ugent.be/resources.

### Ethics

The study protocol was approved by the Ethics Committee of Ghent University Hospital in Belgium (B670201734334) and the ethics committee of Centre Muraz in Burkina Faso (N°2018–22/MS/SG/CM/CEI). An independent Data and Safety Monitoring Board (DSMB), comprising an endocrinologist, 2 pediatricians, a gynecologist, and an ethicist of both Belgian and Burkinabe nationalities, was established before the start of the efficacy trial. The DSMB conducted remote safety reviews for adverse and serious events at 9 and 20 mo after the start of enrollment.

### Statistical analysis

Analyses were recorded in the MISAME-III statistical analysis plan that was validated on 24 October, 2019 and published online on 3 November, 2020 on the study's website: https://www.misame3.ugent.be/resource-files/MISAME-III_SAP_v1_102019.pdf. For consistency and comparability of study findings, the present analyses followed the analytical procedures used to assess the efficacy of the prenatal fortified BEP intervention on birth outcomes (21).

The MISAME-III efficacy trial specified 1 primary prenatal outcome: SGA [<10^th^ percentile of the International Fetal and Newborn Growth Consortium for the 21st Century (INTERGROWTH-21^st^) newborn size standards (35)]. Secondary maternal outcomes of the prenatal BEP intervention included anemia (Hb < 11 g/dL) at the third ANC visit and GWG (kg) between the first and last ANC visits (or before delivery). Furthermore, we estimated the GWG rate, defined as the absolute GWG divided by the time interval between the first and last maternal weight measurements, and the expected weight gain for each woman at the time of their last observed weight measurement using the following IOM 2009 formula (1):

(1) $$
\begin{eqnarray*}
&& {\rm recommended\,\, GWG} = {\rm expected\,\, first-trimester\,\, total\,\, weight\,\, gain}\\
&&\quad + \Big[({\rm GA\,\, at\,\, the\,\, last\,\, weight\,\, measurement})\\
&&\quad - {\rm 13wk\,\, and\,\, 6 d} ({\rm equivalent\,\, to\,\, 13.86 wk})\\
&&\quad \times {\rm recommended\,\, rate\,\, of\,\, GWG\,\, for}\\
&&\quad\quad {\rm the\,\, second\,\, and\,\, third\,\, trimesters\,\, by\,\, BMI\,\, category} \Big]
\end{eqnarray*}
$$

The expected total weight gain during the first trimester was assumed to be 2 kg for underweight (BMI < 18.5) and normal-weight women (BMI = 18.5–24.9), 1 kg for overweight women (BMI = 25–29.9), and 0.5 kg for obese women (BMI ≥ 30); and the recommended rates of GWG for the second and third trimesters were 0.51, 0.42, 0.28, and 0.22 kg/wk for underweight, normal-weight, overweight, and obese women, respectively (1). Finally, the percentage adequacy of GWG was calculated by dividing the actual GWG by the expected GWG at the last observed weight measurement, multiplied by 100. This is a continuous measure that has been used in previous GWG studies in Africa (36, 37). Following Liu et al. (37), we further categorized the percentage adequacy of GWG into binary outcome measures. Inadequate GWG was defined as a percentage adequacy of GWG < 90%, severely inadequate GWG as a percentage adequacy of GWG < 70%, and excessive GWG as a percentage adequacy of GWG ≥ 125%. The cutoffs 90% and 125% correspond to the lower and upper limits of the recommended total weight gain during pregnancy by the IOM's guideline. The recommended range is 12.5–18 kg for women who are underweight (BMI < 18.5), 11.5–16 kg for normal weight (BMI = 18.5–24.9), 7–11.5 kg for overweight (BMI = 25–29.9), and 5–9 kg for obese (BMI ≥ 30) (1).

Only singleton pregnancies were included in the analysis. All analyses were conducted by the intention-to-treat (ITT) principle to reduce potential bias arising from missing data. Therefore, before analyses, we performed multiple imputation of missing maternal Hb concentration (g/dL), maternal weight before delivery (kg), and gestational duration (wk) under the “missing at random” assumption. Fifty imputations of missing continuous outcome data for cases lost to follow-up were run to estimate the regression coefficients, based on the following predictors at baseline: maternal height (cm), maternal weight (kg), MUAC (mm), Hb (g/dL), and age (y) at inclusion; GA at baseline (wk); primiparity; and month of inclusion. Anemia and GWG adequacy variables were calculated from the imputed continuous data.

Descriptive data are presented as percentages or means ± SDs. Unadjusted and adjusted group differences were estimated by fitting linear regression models for the continuous outcomes, to estimate the mean group difference, and using linear probability models with robust variance estimators for the binary outcomes, to estimate risk differences in percentage points (pp). All models were adjusted for the baseline value of the outcome of interest [i.e., either Hb or maternal weight at study enrollment, thus an ANCOVA (38, 39)] and contained health center and randomization block as fixed effects to account for clustering by the study design. Adjusted models in addition contained potential baseline prognostic factors of maternal outcomes, including maternal height (cm), MUAC (mm), and age (y) at inclusion; GA at baseline (wk); and primiparity. We did not adjust for any other sociodemographic variables, owing to balanced baseline characteristics across the prenatal study groups (i.e., < |2.5| pp difference).

To assess the robustness of the primary findings, we conducted the following sensitivity analyses: *1*) complete case analysis (i.e., excluding women who were lost to follow-up for birth outcomes); and *2*) per protocol analysis restricting the intervention sample to women with fortified BEP compliance of ≥75%. The strict compliance rate was calculated by dividing the total number of BEP supplements effectively taken under direct observation of a trained home visitor by the theoretical maximum number of prenatal BEP supplements allowed (i.e., the number of days between study inclusion and delivery). Moreover, to assess the potential underestimation of absolute GWG, we replicated our complete case analysis among mothers who had a baseline weight measurement taken at <14 weeks of gestation (i.e., first trimester). In addition, for complete cases, we used the INTERGROWTH-21^st^ GWG standards to derive GWG *z* scores (40). Because the currently available GWG equation for recommended weight gain is for normal-weight women with a GA between 14 and 40 wk, we restricted this outcome to normal-weight women with a GA of ≤40 completed weeks at their last ANC visit.

Statistical significance was set at *P* < 0.05 for all 2-sided tests. All analyses were conducted with Stata version 16.1 (StataCorp).

## Results

**FIGURE 1:**
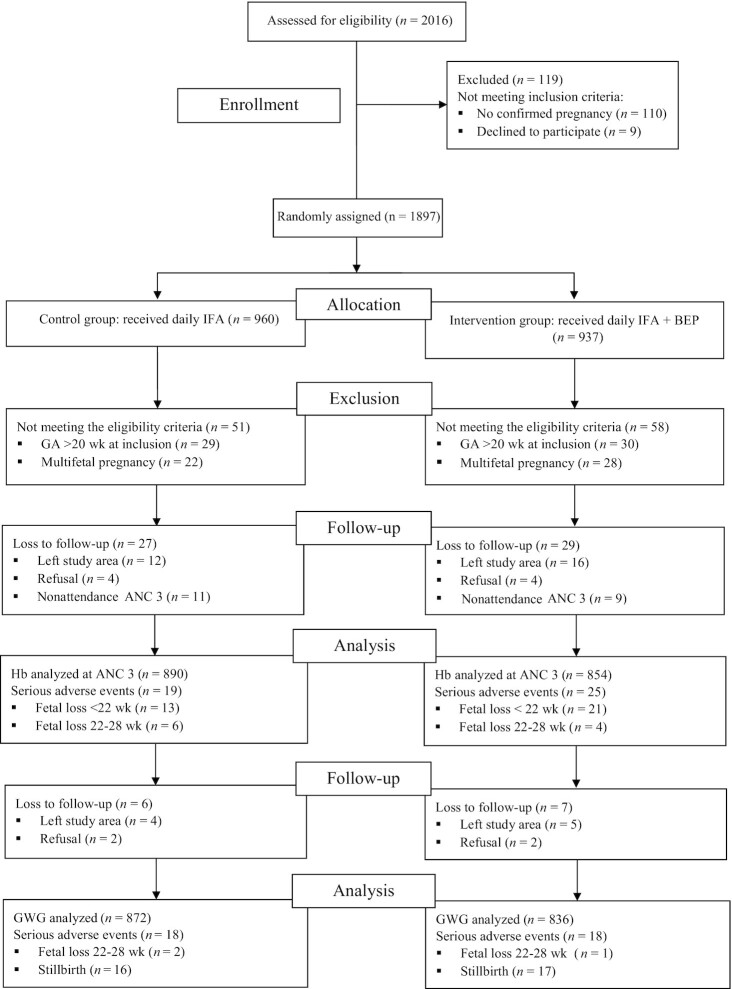
MISAME-III (Micronutriments pour la Santé de la Mère et de l'Enfant study 3) trial flowchart. ANC, antenatal care; BEP, balanced energy-protein; GWG, gestational weight gain; Hb, hemoglobin; IFA, iron–folic acid.

Between October 2019 and December 2020, 2016 women were assessed for eligibility, of whom 1897 were randomly assigned (960 control, 937 intervention) and 119 excluded for not meeting the trial's inclusion criteria. Subsequent ultrasounds, to confirm urine pregnancy test results and to estimate GA, led to postrandomization exclusion of a further 59 women who were at ≥21 weeks of gestation at inclusion and 50 women with a multifetal pregnancy (**Figure 1**). **Table 1** presents the baseline characteristics of mothers included in the ITT analyses of GWG (909 control, 879 intervention). The prenatal trial arms were well balanced regarding household, maternal, and pregnancy characteristics (i.e., < |2.5| pp differences across groups). At baseline, 7.1% of mothers were underweight, 10.8% were overweight, 1.7% were obese, 37.7% were anemic, and the mean ± SD GA was 11.5 ± 4.06 wk. Of the 1788 women with sociodemographic data, 27 control (2.97%) and 29 intervention arm (3.30%) mothers either were lost to follow-up or failed to attend their third ANC visit, whereas in total 22 control (2.42%) and 27 intervention arm mothers (3.07%) were lost to follow-up before delivery (there were 1659 complete cases) (Figure 1).

**TABLE 1:** Baseline characteristics of study participants, by MISAME-III trial arm^1^

Characteristics	IFA (*n* = 909)	IFA + BEP (*n* = 879)
Health center catchment area		
Boni	22.0	21.8
Dohoun	10.5	11.0
Dougoumato II	18.9	17.5
Karaba	10.2	10.7
Kari	18.4	18.7
Koumbia	20.0	20.3
Household		
Asset index (range: 0–10 points)	4.51 ± 1.74	4.67 ± 1.75
Household food insecurity^2^	53.9	55.5
Improved primary water source^3^	62.2	62.7
Improved sanitation facility^4^	59.3	60.6
Household size, *n*	6.19 ± 4.45	6.20 ± 4.21
Polygamous households	31.8	32.7
Head of household		
Age, y	33.4 ± 9.16	33.8 ± 9.33
Male	99.7	99.8
Completed primary education	59.8	59.2
Maternal		
Age, y	25.1 ± 6.20	25.0 ± 6.18
Ethnic group		
Bwaba	57.3	57.6
Mossi	35.3	34.5
Other	7.37	7.96
Religion		
Muslim	42.1	42.3
Animist	23.4	22.8
Protestant	16.2	18.4
Catholic	14.4	13.1
No religion, no animist	3.85	3.19
Completed primary education	42.4	41.4
Weight, kg	57.9 ± 8.65	58.4 ± 8.69
Height, cm	162 ± 5.91^5^	163 ± 6.05
BMI, kg/m^2^	22.0 ± 2.87	22.0 ± 2.87
<18.5	7.05	7.17
25–29.9	10.3	11.3
≥30	1.54	1.82
Midupper arm circumference, mm	262 ± 26.8	262 ± 26.4
Subscapular skinfold, mm	11.9 ± 5.47	12.1 ± 5.58
Tricipital skinfold, mm	11.8 ± 4.76	12.0 ± 4.86
Hb, g/dL	11.4 ± 1.47	11.3 ± 1.52
Anemia (Hb < 11 g/dL)	36.7	38.7
Severe anemia (Hb < 7 g/dL)	0.22	0.23
Gestational age, wk	11.4 ± 4.08	11.5 ± 4.04
Trimester of gestation		
First	63.1	62.0
Second	36.9	38.0
Parity		
0	21.8	23.1
1–2	35.9	33.4
≥3	42.4	43.5

1 Values are percentages or means ± SDs. BEP, balanced energy-protein; Hb, hemoglobin; IFA, iron–folic acid; MISAME-III, Micronutriments pour la Santé de la Mère et de l'Enfant study 3.

2 Assessed using Food and Nutrition Technical Assistance Project (FANTA)/USAID's Household Food Insecurity Access Scale (72).

3 Protected well, borehole, pipe, or bottled water were considered improved water sources.

4 Flush toilet connected to local sewage or septic tank, or pit latrine with slab and/or ventilation were considered as improved sanitation facilities.

5 The height of 1 woman with a physical disability could not be measured.

The third ANC visit was completed at (mean ± SD) 26.6 ± 3.43 and 26.4 ± 3.36 weeks of gestation for women in the intervention and control groups, respectively. Our unadjusted ITT analyses (3.21% of observations were imputed) of a combined daily BEP supplement and IFA tablet indicated a nonsignificant difference in maternal Hb concentration (0.02 g/dL; 95% CI: −0.09, 0.14 g/dL; *P* = 0.701), anemia (1.01 pp; 95% CI: −3.60, 5.60 pp; *P* = 0.665), or severe anemia prevalence (0.12 pp; 95% CI: −0.11, 0.35 pp; *P* = 0.319) at ANC 3, as compared with an IFA tablet alone (**Table 2**). These findings were confirmed (*P* > 0.05) by adjusting the regression models for prognostic factors of maternal anemia at enrollment (Table 2), by complete case analyses (**Supplemental Table 2**), and by per protocol analyses (**Supplemental Table 3**).

**TABLE 2:** Efficacy of prenatal fortified BEP supplementation on maternal outcomes^1^

Women's characteristics	IFA (*n* = 890)	IFA + BEP (*n* = 854)	Unadjusted ∆ (95% CI)	*P*	Adjusted ∆ (95% CI)	*P*
Hb at ANC 3, g/dL	10.9 ± 1.28	11.0 ± 1.24	0.02 (−0.09, 0.14)^2^	0.701	0.03 (−0.08, 0.14)^2^	0.604
Anemia (Hb < 11 g/dL) at ANC 3	47.8	49.4	1.01 (−3.60, 5.60)^2^	0.665	0.67 (−3.89, 5.23)^2^	0.774
Severe anemia (Hb < 7 g/dL) at ANC 3	0	0.12	0.12 (−0.11, 0.35)^2^	0.319	0.11 (−0.11, 0.33)^2^	0.321
GWG,^3^ kg	6.00 ± 3.52	6.27 ± 3.52	0.28 (−0.05, 0.60)^2^	0.099	0.27 (−0.05, 0.58)^2^	0.095
GWG rate,^3^ kg/wk	0.261 ± 0.155	0.274 ± 0.189	0.013 (−0.004, 0.030)^2^	0.141	0.013 (−0.004, 0.029)^2^	0.128
GWG adequacy,^3,4^ %	57.0	60.0	2.88 (−0.84, 6.60)^5^	0.129	3.09 (−0.60, 6.78)^5^	0.101
Inadequate GWG (<90%)^3,4^	86.2	83.4	−2.81 (−6.20, 0.57)^5^	0.103	−2.97 (−6.34, 0.40)^5^	0.084
Severely inadequate GWG (<70%)^3,4^	68.3	64.7	−3.43 (−7.87, 1.01)^5^	0.130	−3.72 (−8.12, 0.68)^5^	0.097
Excessive GWG (≥125%)^3,4^	3.05	4.23	1.15 (−0.63, 2.93)^5^	0.205	1.19 (−0.57, 2.95)^5^	0.184

1 Values are percentages or means ± SDs unless otherwise indicated. ANC, antenatal care; BEP, balanced energy-protein; GWG, gestational weight gain; Hb, hemoglobin; IFA, iron–folic acid; MUAC, midupper arm circumference.

2 Unadjusted and adjusted group differences were estimated by fitting linear regression models for the continuous outcomes, to estimate the mean group difference, and using linear probability models with robust variance estimation for the binary outcomes, to estimate risk differences in percentage points. All models were adjusted for the baseline outcome [i.e., Hb (g/dL) or weight (kg)] and contained health center and randomization block as fixed effects to account for clustering by the study design. Adjusted models in addition contained a set of a priori–determined known prognostic factors of outcomes including maternal age, primiparity, gestational age, height, and MUAC at study enrollment.

3 *n* = 872 for IFA group GWG measures; *n* = 836 for IFA + BEP group GWG measures.

4 Expected GWG gain during the first trimester was assumed to be 2 kg for underweight [BMI (in kg/m^2^) <18.5] and normal-weight women (BMI = 18.5–24.9), 1 kg for overweight women (BMI = 25–29.9), and 0.5 kg for obese women (BMI ≥ 30); and the recommended rates of GWG for the second and third trimesters were 0.51, 0.42, 0.28, and 0.22 kg/wk for underweight, normal-weight, overweight, and obese women, respectively. GWG adequacy percentage was calculated by dividing the actual GWG by the expected GWG at the last observed weight measurement, then multiplying by 100.

5 Unadjusted and adjusted group differences were estimated by fitting linear regression models for continuous GWG adequacy, to estimate the mean group difference, and using linear probability models with robust variance estimation for the binary GWG adequacy outcomes, to estimate risk differences in percentage points. All models contained health center and randomization block as fixed effects to account for clustering by the study design. Adjusted models in addition contained a set of a priori–determined known prognostic factors of outcomes including maternal age, primiparity, and MUAC at study enrollment.

Furthermore, the last maternal weight measurement was taken at (mean ± SD) 35.5 ± 4.35 weeks of gestation in the combined BEP and IFA arm and at 35.6 ± 4.51 weeks of gestation for the IFA-only arm. Our unadjusted ITT analyses (2.87% of observations were imputed) did not show a significant difference across study arms for total GWG (0.28 kg; 95% CI: −0.05, 0.60 kg; *P* = 0.099), GWG rate (13 g/wk; 95% CI: −4, 30 g/wk; *P* = 0.141), GWG adequacy (2.88 pp; 95% CI: −0.84, 6.60 pp; *P* = 0.129), and inadequate GWG (−2.81 pp; 95% CI: −6.20, 0.57 pp; *P* = 0.103), severely inadequate GWG (−3.43 pp; 95% CI: −7.87, 1.01 pp; *P* = 0.130), or excessive GWG prevalence (1.15 pp; 95% CI: −0.63, 2.93 pp; *P* = 0.205) (Table 2). Our main findings were confirmed (*P* > 0.05) by adjusting the regression model for prognostic factors of maternal GWG at baseline (Table 2) and per protocol analysis (Supplemental Table 3). Nevertheless, complete case analyses indicated small, but significant, differences in absolute GWG and GWG adequacy (Supplemental Table 2). Restricting our analysis to only women with a baseline weight measurement in the first trimester of gestation did not change our results. To summarize, mean ± SD absolute GWG was 6.25 ± 3.62 kg in the control arm (*n* = 600) and 6.56 ± 3.64 kg (*n* = 583) in the intervention arm; and the unadjusted mean difference was 0.30 kg (95% CI: −0.09, 0.71 kg; *P* = 0.143).

Lastly, among women with a normal BMI at baseline and a GA ≤ 280 d at their last weight measurement, the mean ± SD of GWG *z* scores were −1.52 ± 1.26 SD in the IFA arm (*n* = 584) and −1.48 ± 1.27 SD in the fortified BEP and IFA arm (*n* = 524); and the unadjusted mean difference was 0.05 SD (95% CI: −0.09, 0.21 SD; *P* = 0.480).

## Discussion

In the MISAME-III trial, we found that pregnant women who received a daily fortified BEP supplement and IFA tablet did not have different (midline) Hb concentrations, total GWG, GWG rates, or prevalence of (severely) inadequate GWG and excessive GWG, as compared with those women who received an IFA tablet only.

The absence of an increase in prenatal Hb and GWG in the BEP group was unexpected (41), given that the mean energy and iron contents of the fortified BEP supplement were 393 kcal/d and 22 mg/d (and mean BEP and IFA compliance were both >80%), respectively (21). The lack of efficacy on Hb concentration might be explained by the BEP's zinc (15 mg/d) (42) and calcium (500 mg/d) competing with or blocking the mucosal uptake of iron in the gut (43), respectively. Another clarification might be that the maximum Hb response was already achieved by the elemental iron (∼22.4 mg/d) intake from the IFA tablets over follow-up (44). Moreover, despite all women receiving daily IFA tablets, anemia prevalence in fact increased on average by ∼10 pp during pregnancy, which is probably due to physiologic hemodilution in the second trimester (45).

A cross-sectional substudy in MISAME-III indicated that BEP supplementation increased energy and macro- and micronutrient intakes and filled nutrient gaps without displacing food intakes among pregnant women (27). Correspondingly, a longitudinal follow-up study in MISAME-III reported that usual prenatal dietary diversity did not differ across trial arms (25). Hence, it is unlikely that the observed null effects on Hb and GWG are due to BEP supplements displacing nutrient or food group intakes. The effect of macronutrient supplementation is nevertheless dependent on prior maternal energy deficits, whereas >90% of mothers in MISAME-III were normal weight or overweight (including obese) at enrollment and thus potentially not (sufficiently) vulnerable to macronutrient deficiencies [i.e., WHO antenatal care guidelines suggest use of BEP where the population-level prevalence of low BMI (<18.5) is >20%] (2).

Limited research has examined the effectiveness or efficacy of prenatal MMN or (fortified) BEP supplementation on GWG, and the results are heterogeneous (16). Our findings were similar to maternal supplementation interventions administering either SQ-LNSs in Bangladesh [mean GWG rate: 0.29 kg/wk (control) compared with 0.30 kg/wk] (46), Corn Soya Blend in Cambodia (mean GWG: 8.1 kg compared with 8.5 kg) (47), or an LNS and fortified tea in The Gambia (mid- and late-pregnancy GWG differences: both *P* > 0.05) (48). Similarly, an RCT in Ghana reported that there were no differences in GWG measures between pregnant women who received either SQ-LNSs or MMN supplements and those who received IFA supplements (e.g., mean GWG: 7.2 kg or 7.7 kg compared with 7.3 kg) (36). Moreover, an RCT conducted in Mexico reported that MMN supplements did not increase weight gain during pregnancy when compared with iron supplements alone (mean GWG: 7.6 kg compared with 7.3 kg) (49). In contrast, 2 large RCTs conducted in Tanzania among HIV-negative women (50) and HIV-infected women (51) demonstrated that prenatal MMN supplements were significantly associated with a 253-g (95% CI: 177, 388 g) and 304-g (95% CI: 17, 590 g) greater total (and third-trimester) GWG than placebo, respectively. Moreover, using the IOM's recommendations, the GWG adequacy difference between the MMN and placebo arms was 2.3 pp (95% CI: 0.3, 4.2 pp) (37). In MISAME-III, we enrolled a substantially smaller sample of pregnant women than did Liu et al. (37) (*n* = 7573); however, our BEP intervention also indicated mean increases of similar magnitudes for absolute GWG and GWG adequacy, i.e., 276 g (95% CI: −5.4, 597 g) and 2.9 pp (−0.84, 6.6 pp), respectively. Furthermore, when a prenatal MMN-fortified milk-based product was compared against a nonfortified powdered milk in Chile, an increase in maternal weight gain was also observed (mean GWG: 12.3 kg compared with 11.3 kg) (52). Correspondingly, a BEP intervention in Thailand concluded that providing 13 g protein and 350 kcal during the third trimester significantly improved maternal weight gain (mean GWG rate: 0.45 kg/wk compared with 0.28 kg/wk) (53). In the multicountry Women First trial, preconception SQ-LNS supplementation increased maternal weight gain, as compared with the same supplement commenced late in the first trimester of pregnancy or not at all (mean GWG: 6.9 kg compared with 6.4 kg compared with 6.2 kg) (54). The discrepancies between trial findings might be due to the differences in study population and intervention doses and timing (16). To illustrate, MMN trials in Nepal and Bangladesh have brought up the concern that single RDA regimens may be suboptimal in settings of widespread undernutrition and have not resulted in micronutrient deficiency corrections (42, 55).

Our current result that fortified BEP supplementation was not significantly associated with improvements in measures of GWG (i.e., potential mediating factor) is consistent with the previously reported MISAME-III finding of modest effects on anthropometric birth outcomes (21). Overall, an estimated 50% of absolute GWG is ascribed to the feto-placental unit; 25% to blood volume expansion, extravascular fluid, and breast tissue; and the remaining 25% to maternal fat stores (1). Early pregnancy weight gain is slow and primarily due to maternal fat deposition, total body water accretion, and placental and other tissue development, whereas later pregnancy weight gain is thought to be more related to fetal growth (1). Previous studies have suggested that GWG during the first and second trimesters has a stronger effect on birth weight than GWG that occurs in the third trimester (56). Nevertheless, GWG in late pregnancy was associated with higher placental and birth weights in rural Bangladesh (57).

In addition, we report that GWG was ∼6 kg in both intervention and control arms, ∼85% of the study participants experienced inadequate GWG, and ∼65% experienced severely inadequate GWG during pregnancy. This finding is consistent with a recent meta-analysis of studies from sub-Saharan Africa, in which the authors reported that the percentage of inadequate GWG, as defined according to the IOM's recommendation, was >60% in 8 of the 16 studies (6). Furthermore, observational studies in rural Malawi (58), Niger (59), Bangladesh (60, 61), and peri-urban India (62) reported that 72%, 63%, 74% and 54%, and 40% of pregnant women had inadequate GWG, respectively, whereas RCTs conducted in Ghana (36), Vietnam (63), and Tanzania (37) reported that 63%, 62%, and ∼50% of mothers experienced inadequate GWG, respectively. Nonetheless, in MISAME-III only ∼7% and ∼12% of women were underweight and overweight (including obese) at baseline, respectively, which is substantially lower than most GWG studies conducted in sub-Saharan Africa (6). We therefore hypothesize that in LMICs, inadequate GWG might be more prevalent among normal-weight women, because the range of the IOM's recommendation for these mothers is slightly narrower than for underweight women, and the minimum acceptable GWG is greater than for overweight and obese women (1).

The current study has several strengths. First, MISAME-III used an individually randomized design with an IFA control group (i.e., the standard of care). Second, we examined the effect of BEP supplements on absolute GWG, GWG rate, and GWG in relation to the IOM's recommendations. In contrast, previous epidemiologic studies of GWG have often used either total GWG in kilograms or mean GWG rate only, which may be biased owing to their correlation with gestation duration (64). By including the IOM's adequacy ratios and INTERGROWTH-21^st^ GWG *z* scores (by definition independent of pregnancy length), among normal-weight mothers the observed (nonsignificant) small increase in total GWG in the fortified BEP arm could be explained independently from the supplement's known efficacy on gestational duration (i.e., on average 1.4 d, translating to an ∼84-g increase among normal-weight women) (21). Third, in our RCT, GA was determined using ultrasound, the gold-standard method, rather than the error-prone last menstrual period (e.g., recall bias, irregular menses) (62).

However, our study has some limitations that warrant caution. First, the efficacy of prenatal BEP supplementation on third-trimester anemia prevalence (i.e., when RBC volume is more proportional to the hydremia of pregnancy) could not be assessed, because maternal Hb concentrations were not measured before delivery. Second, the IOM's recommendations might still lead to misclassification if women gain well above or below the assumed first-trimester weight gain (65). Third, because prepregnancy BMI is one of the main determinants of GWG, findings on total weight gain or GWG rate from different studies might not be comparable if there were large differences in prepregnancy BMI across studies (4, 66). Fourth, because this RCT enrolled participants after their pregnancies were confirmed, prepregnancy BMI was not available in this study. Therefore, because prepregnancy BMI is required to calculate the recommended weight gain based on the IOM and INTERGROWTH-21^st^ recommendations, we used BMI measured in the late first or early second trimester (the latter excluded in the sensitivity analyses) as a substitute for prepregnancy BMI given that weight gain during the first trimester is minimal (67). Fifth, the last maternal weight measurement was taken at ∼36 weeks of gestation, rather than before delivery (at ∼40 weeks of gestation); hence total GWG, and subsequently GWG adequacy, were likely underestimated. We believe that the potential measurement error for GWG is not differential across study arms, resulting in unbiased results for efficacy. Sixth, our use of total pregnancy weight gain measures to summarize GWG is not sensitive to a mother's weight gain pattern/timing (68) or more granular changes in maternal body composition (e.g., fat-free mass) (57). Lastly, the hypotheses that increased GWG might reflect lower amounts of physical work during pregnancy in LMICs (69) or lower (subclinical) inflammation and greater placental angiogenesis as a consequence of BEP (70, 71), and thus better fetal nourishment, could not be tested because neither maternal physical activity, nor biological markers, were measured at baseline in the MISAME-III trial.

In conclusion, our results indicate that the provision of daily fortified BEP supplements to pregnant women did not improve absolute GWG, GWG rate, GWG in relation to the IOM's recommendations, or GWG *z* scores. Future randomized interventions might assess whether (preconception) environments conducive to adequate GWG allow the mother to be more nutritionally replete, permitting any additional nutrients from supplementation to support fetal growth and development.

## Supplementary Material

nxac171_Supplemental_File

## Data Availability

The informed consent form does not allow sharing of personal data outside the research team. Requests to access data need to be directed to the ethics committee of Ghent University Hospital through ethisch.comite@uzgent.be. Supporting study documents, including the study protocol and questionnaires, are publicly available on the study's website: https://misame3.ugent.be.

## Notes

Supported by Bill & Melinda Gates Foundation grant OPP1175213 (to PK). The funder had no role in study design, data collection and analysis, the decision to publish, or preparation of the manuscript.

Author disclosures: The authors report no conflicts of interest.

Supplemental Tables 1–3 are available from the “Supplementary data” link in the online posting of the article and from the same link in the online table of contents at https://academic.oup.com/jn/.

Abbreviations used: ANC, antenatal care; BEP, balanced energy-protein; DSMB, Data and Safety Monitoring Board; EAR, Estimated Average Requirement; GA, gestational age; GWG, gestational weight gain; Hb, hemoglobin; IFA, iron–folic acid; INTERGROWTH-21st, International Fetal and Newborn Growth Consortium for the 21st Century; IOM, Institute of Medicine; ITT, intention-to-treat; LMIC, low- and middle-income country; LNS, lipid-based nutrient supplement; MISAME, MIcronutriments pour la SAnté de la Mère et de l'Enfant; MMN, multiple micronutrient; MUAC, midupper arm circumference; pp, percentage point; RCT, randomized controlled trial; SGA, small-for-gestational age; SQ-LNS, small-quantity lipid-based nutrient supplement.
