# Supplementary material for: Fortified Balanced Energy-Protein Supplementation, Maternal Anemia, and Gestational Weight Gain: A Randomized Controlled Efficacy Trial among Pregnant Women in Rural Burkina Faso
Source: J Nutr. 2022 Jul 30;152(10):2277–86. doi: 10.1093/jn/nxac171 (PMC9535447; doi:10.1093/jn/nxac171)
Supplement: nxac171_Supplemental_File [file nxac171_supplemental_file.docx]

**Supplemental Table 1. Nutritional values of the ready-to-use supplementary food for pregnant and lactating women^1^**

|  | Mean for 72g (serving size) |
| --- | --- |
| Total energy (kcal) | 393 |
| Lipids (g) | 26 |
| Linoleic acid (g) | 3.9 |
| α-Linoleic acid (g) | 1.3 |
| Proteins (g) | 14.5 |
| Carbohydrates (g) | 23.3 |
| Calcium (mg) | 500 |
| Copper (mg) | 1.3 |
| Phosphorus (mg) | 418 |
| Iodine (*µ*g) | 250 |
| Iron (mg) | 22 |
| Selenium (*µ*g) | 65 |
| Manganese (mg) | 2.1 |
| Magnesium (mg) | 73 |
| Potassium (mg) | 562 |
| Zinc (mg) | 15 |
| Vitamin A (*µ*g RE)^2^ | 770 |
| Thiamin (mg) | 1.4 |
| Riboflavin (mg) | 1.4 |
| Niacin (mg) | 15 |
| Vitamin B5 (mg) | 7 |
| Vitamin B6 (mg) | 1.9 |
| Folic acid (*µ*g) | 400 |
| Vitamin B12 (mg) | 2.6 |
| Vitamin C (mg) | 100 |
| Vitamin D (*µ*g cholecalciferol)^3^ | 15 |
| Vitamin E (mg α-tocopherol)^4^ | 18 |
| Vitamin K (*µ*g) | 72 |

^1^Ingredients: vegetable oils (rapeseed, palm, soy in varying proportions), defatted soy flour, skimmed milk powder, peanuts, sugar, maltodextrin, soy protein isolate, vitamin and mineral complex, stabilizer (fully hydrogenated vegetable fat, mono- and diglycerides). IU, international unit, RE, retinol equivalent.

^2^1 *µ*g vitamin A RE = 3.333 IU vitamin A.

^3^1 *μ*g cholecalciferol = 40 IU vitamin D.

^4^1 mg α-tocopherol = 2,22 IU vitamin E.

**Supplemental Table 2. Complete cases analysis of maternal prenatal outcomes**

| **Women’s characteristics** | **IFA^1^**  **(*n*=863)** | **IFA + BEP^1^**  **(*n*=825)** | **Unadjusted ∆**  **(95% CI)** | ***P*** | **Adjusted ∆**  **(95% CI)** | ***P*** |
| --- | --- | --- | --- | --- | --- | --- |
| Hb at ANC 3, g/dL | 11.0 ± 1.28 | 11.0 ± 1.24 | 0.02 (-0.09, 0.13)^2^ | 0.731 | 0.03 (-0.09, 0.14)^2^ | 0.650 |
| Anemia (Hb <11g/dL) at ANC 3 | 47.5 | 49.3 | 1.10 (-3.50, 5.69)^2^ | 0.640 | 0.89 (-3.67, 5.44)^2^ | 0.703 |
| Severe anemia (Hb <7g/dL) at ANC 3 | 0.00 | 0.12 | 0.12 (-0.11, 0.34)^2^ | 0.319 | 0.11 (-0.10, 0.32)^2^ | 0.320 |
|  | **(*n*=850)** | **(*n*=809)** |  |  |  |  |
| GWG, kg | 6.02 ± 3.53 | 6.33 ± 3.50 | 0.33 (0.00, 0.66)^2^ | 0.049 | 0.32 (0.00, 0.64)^2^ | 0.047 |
| GWG rate, kg/week | 0.258 ± 0.154 | 0.273 ± 0.163 | 0.014 (-0.001, 0.029) | 0.073 | 0.014 (0.000, 0.028) | 0.055 |
| GWG adequacy, %^3^ | 56.6 | 60.8 | 4.14 (0.63, 7.64)^4^ | 0.021 | 4.37 (0.90, 7.85)^4^ | 0.014 |
| Inadequate GWG (<90%) | 86.2 | 83.3 | -2.93 (-6.36, 0.49)^4^ | 0.093 | -3.12 (-6.51, 0.30)^4^ | 0.074 |
| Severely inadequate GWG (<70%) | 68.5 | 64.8 | -3.60 (-8.08, 0.88)^4^ | 0.115 | -3.92 (-8.36, 0.52)^4^ | 0.083 |
| Excessive GWG (≥125%) | 3.06 | 4.20 | 1.17 (-0.63, 2.97)^4^ | 0.203 | 1.22 (-0.56, 3.00)^4^ | 0.178 |

^1^Values are percentages or means ± SDs, excluding women who were lost to follow-up for birth outcomes. ANC, antenatal care; BEP, balanced energy-protein; GWG, gestational weight gain; Hb, hemoglobin; IFA, iron-folic acid.

^2^Unadjusted and adjusted group differences were estimated by fitting linear regression models for the continuous outcomes, to estimate the mean group difference, and using linear probability models with robust variance estimation for the binary outcomes, to estimate risk difference in percentage points. All models were adjusted for baseline outcome [i.e., Hb (g/dL) or weight (kg)] and contained health center and randomization block as fixed effects to account for clustering by the study design. Adjusted models additionally contained a set of *a priori* determined known prognostic factors of outcomes including maternal age, primiparity, gestational age, height, and mid-upper arm circumference at study enrolment.

^3^Expected GWG gain during the first trimester was assumed to be 2 kg for underweight (BMI <18.5 kg/m²) and normal weight women (BMI 18.5 to 24.9 kg/m²), 1 kg for overweight women (BMI 25 to 29.9 kg/m²), and 0.5 kg for obese women (BMI ≥30 kg/m²); and the recommended rates of GWG for the second and third trimesters were 0.51, 0.42, 0.28, and 0.22 kg/week for underweight, normal weight, overweight, and obese women, respectively. GWG adequacy percentage was calculated by dividing the actual GWG by the expected GWG at the last observed weight measurement, multiplied by 100.

^4^Unadjusted and adjusted group differences were estimated by fitting linear regression models for continuous GWG adequacy, to estimate the mean group difference, and using linear probability models with robust variance estimation for the binary GWG adequacy outcomes, to estimate risk difference in percentage points. All models contained health center and randomization block as fixed effects to account for clustering by the study design. Adjusted models additionally contained *a priori* set known prognostic factors of outcomes including maternal age, primiparity, and mid-upper arm circumference at study enrolment.

**Supplemental Table 3. Per protocol analysis of maternal prenatal outcomes**

| **Women’s characteristics** | **IFA^1^**  **(*n*=863)** | **IFA + BEP1^1,2^**  **(*n*=641)** | **Unadjusted ∆**  **(95% CI)** | ***P*** | **Adjusted ∆**  **(95% CI)** | ***P*** |
| --- | --- | --- | --- | --- | --- | --- |
| Hb at ANC 3, g/dL | 11.0 ± 1.28 | 11.0 ± 1.22 | 0.03 (-0.09, 0.16)^3^ | 0.593 | 0.03 (-0.09, 0.15)^3^ | 0.581 |
| Anemia (Hb <11g/dL) at ANC 3 | 47.5 | 49.5 | 1.48 (-3.43, 6.40)^3^ | 0.553 | 1.46 (-3.41, 6.32)^3^ | 0.557 |
| Severe anemia (Hb <7g/dL) at ANC 3 | 0.00 | 0.00 | - | - | - | - |
|  | **(*n*=850)** | **(*n*=631)** |  |  |  |  |
| GWG, kg | 6.02 ± 3.53 | 6.26 ± 3.42 | 0.28 (-0.08, 0.63)^3^ | 0.125 | 0.27 (-0.06, 0.61)^3^ | 0.113 |
| GWG rate, kg/week | 0.258 ± 0.154 | 0.272 ± 0.166 | 0.014 (-0.003, 0.030) | 0.098 | 0.014 (-0.001, 0.030) | 0.068 |
| GWG adequacy, %^4^ | 56.6 | 59.9 | 3.48 (-0.24, 7.20)^5^ | 0.066 | 3.64 (-3.96, 7.33)^5^ | 0.053 |
| Inadequate GWG (<90%) | 86.2 | 83.5 | -2.78 (-6.48, 0.91)^5^ | 0.139 | -2.84 (-6.52, 0.85)^5^ | 0.131 |
| Severely inadequate GWG (<70%) | 68.5 | 65.8 | -3.00 (-7.84, 1.83)^5^ | 0.223 | -3.14 (-7.91, 1.64)^5^ | 0.198 |
| Excessive GWG (≥125%) | 3.06 | 3.65 | 0.44 (-1.41, 2.28)^5^ | 0.643 | 0.37 (-1.44, 2.20)^5^ | 0.684 |

^1^Values are percentages or means ± SDs. ANC, antenatal care; BEP, balanced energy-protein; GWG, gestational weight gain; Hb, hemoglobin; IFA, iron-folic acid.

^2^Subsample of women meeting a strict compliance rate ≥75%. Strict compliance was defined as the number of days with observed BEP supplement intake over the total days between study inclusion and delivery.

^3^Unadjusted and adjusted group differences were estimated by fitting linear regression models for the continuous outcomes, to estimate the mean group difference, and using linear probability models with robust variance estimation for the binary outcomes, to estimate risk difference in percentage points. All models were adjusted for baseline outcome [i.e., Hb (g/dL) or weight (kg)] and contained health center and randomization block as fixed effects to account for clustering by the study design. Adjusted models additionally contained a set of *a priori* determined known prognostic factors of outcomes including maternal age, primiparity, gestational age, height, and mid-upper arm circumference at study enrolment.

^4^Expected GWG gain during the first trimester was assumed to be 2 kg for underweight (BMI <18.5 kg/m²) and normal weight women (BMI 18.5 to 24.9 kg/m²), 1 kg for overweight women (BMI 25 to 29.9 kg/m²), and 0.5 kg for obese women (BMI ≥30 kg/m²); and the recommended rates of GWG for the second and third trimesters were 0.51, 0.42, 0.28, and 0.22 kg/week for underweight, normal weight, overweight, and obese women, respectively. GWG adequacy percentage was calculated by dividing the actual GWG by the expected GWG at the last observed weight measurement, multiplied by 100.

^5^Unadjusted and adjusted group differences were estimated by fitting linear regression models for continuous GWG adequacy, to estimate the mean group difference, and using linear probability models with robust variance estimation for the binary GWG adequacy outcomes, to estimate risk difference in percentage points. All models contained health center and randomization block as fixed effects to account for clustering by the study design. Adjusted models additionally contained *a priori* set known prognostic factors of outcomes including maternal age, primiparity, and mid-upper arm circumference at study enrolment.
